# Supplementary material for: Co-expression in tissue-specific gene networks links genes in cancer-susceptibility loci to known somatic driver genes
Source: BMC Med Genomics. 2024 Jul 15;17:186. doi: 10.1186/s12920-024-01941-4 (PMC11247850; doi:10.1186/s12920-024-01941-4)
Supplement: Supplementary file 1 — Supplementary Material 1 [file 12920_2024_1941_MOESM1_ESM.pdf]

Supplementary data

Note S1. Quality control and sample selection of recount3 data
Note S2. Tissue prediction and per tissue quality control of recount3 data
Fig. S1. Number of cancer-specific drivers and the number shared across tumour types
Fig. S2. Enrichment of cancer-specific driver genes for different PascalX prioritisations
Fig. S3. Enrichment of cancer-specific somatic and germline driver genes for different multi-tissue- and tissue-specific-based gene-prioritisation methods applied to cancer GWAS summary statistics
Fig. S4. Enrichment of cancer-specific driver genes for different tissue-specific matched networks
Fig. S5. Enrichment of cancer-specific somatic driver genes for all combinations of different multi-tissue and tissue-specific gene-prioritisation methods applied to cancer GWAS summary statistics using PoPS.
Fig. S6. Enrichment of COSMIC tier 1 cancer-specific driver genes for different tissue-specific matched networks
Fig. S7. Enrichment of cancer drivers suspected to be tumour suppressors
Fig. S8. Enrichment of cancer drivers suspected to be oncogenes
Fig. S9. Enrichment of cancer drivers exclusive to their tissue of origin for the different prioritisation scores
Fig. S10. Correlations of Downstreamer Z-scores of all combinations of different multi-tissue and tissue-specific networks
Fig. S11. Downstreamer gene-prioritisation scores from multi-tissue networks versus loss-of-function Z-scores indicating gene constraint
Fig. S12. Downstreamer gene-prioritisation scores from tissue-specific networks versus loss-of-function Z-scores indicating gene constraint
Table S1. All GWAS of cancer traits considered in this study
Table S2. Somatic cancer driver genes
Table S3. PascalX scores
Table S4. Downstreamer gene prioritisation
Table S5. Gene linkage disequilibrium scores
Data S1. Downstreamer gene prioritization (flat text)
Data S2. Pathway analysis

Note S1. *Quality control and sample selection of recount3 data*

Phase 1: We first performed a rough selection of samples using the following steps.

- Remove samples annotated as single cell (n=74,412).
- Remove 4SU-labelled samples (n=1,589).
  - Mention of '4su' or 'thiouridine' in one of these columns:

    "sra.library_construction_protocol"; "sra.study_abstract"; "sra.experiment_title";

"sra.design_description"; "sra.sample_description";

"sra.library_construction_protocol"; "sra.sample_attributes"; "sra.sample_title"

- Remove samples with only NaN expression values (n=239).
- Remove samples with missing metadata (n=1,711).
- Exclude samples based on the following quality control (QC) metrics (n=96,597):
    - sra.sample_spots   <1e6 or >2e8
        - for TCGA samples, recount_qc.bc_frag.count   <1e6 or >2e8
    - recount_qc.star.uniquely_mapped_reads_%   <60%
    - recount_qc.aligned_reads%.chrm   >20%
    - recount_qc.aligned_reads%.chrx   >6%
    - recount_qc.aligned_reads%.chry   >0.5%
    - recount_seq_qc.%n   >2%
    - recount_seq_qc.%a   <20% or >35%
    - recount_seq_qc.%c   <20% or >35%
    - recount_seq_qc.%g   <20% or >35%
    - recount_seq_qc.%t   <20% or >35%
    - recount_qc.star.%_of_reads_mapped_to_too_many_loci   >0.5%
    - recount_qc.junction_count   >500,000
    - recount_qc.star.deletion_average_length   >3
    - recount_qc.star.number_of_splices:_total   <150,000
    - recount_qc.intron_sum_%   >20
    - recount_qc.bc_auc.unique_%   <125
- Exclude all data from study SRP025982 (mixed tissues and spiked data for benchmarks).

Phase 2: We only retained genes that were expressed in at least 50% of the samples.

Phase 3: We performed another sample QC using only the maintained genes.

- Exclude samples with 0 expression >50% of the genes.
- Remove duplicate samples.
- Exclude samples with 0 variance.
- Use singular value decomposition (SVD) on quantile-normalised expression to remove outliers on the first component.

Phase 4: We corrected the remaining samples for covariates using the following steps.

- Correct the expression data for the following technical covariates:
    - recount_seq_qc.avg_len
    - sra.sample_spots
        - recount_qc.bc_frag.count
    - recount_qc.star.uniquely_mapped_reads_%
    - sra.library_layout
    - recount_qc.aligned_reads%.chrm
    - recount_qc.aligned_reads%.chrx
    - recount_qc.aligned_reads%.chry
    - recount_seq_qc.%a
    - recount_seq_qc.%c
    - recount_seq_qc.%g
    - recount_seq_qc.%t
    - recount_qc.bc_auc.unique_%
    - recount_qc.intron_sum_%

- recount_qc.star.%_of_reads_mapped_to_too_many_loci
- recount_qc.junction_count
- recount_qc.star.deletion_average_length

- 675 SRA samples were excluded for missing covariate data. Thus, the total number of samples included was 142,849.

Phase 5: We predicted cell lines and cancer samples. The predictions were based on the sample principal components and trained using the annotations known for a subset of the samples. For the prediction of primary tissues vs cell lines, we used logistic regression using the principal components.

For the prediction of cancer samples, we used the method developed by Fehrmann *et al.* [1]. This first determines the auto-correlation per component, which is higher for components that reflect copy number alterations. The sample loadings are then used to create a score per sample that indicates the amount of copy number alterations in the samples. We could then use this score in a second logistic regression model that discriminated between primary tissues and cancer samples.

Neither of these models yielded perfect separation between the three classes of samples. While this is in part driven by erroneous annotations in the public repositories, further QC could improve the creation of the tissue-specific subsets.

Note S2. *Tissue prediction and per tissue quality control of recount3 data*

To predict tissues for the samples that are predicted to not be cell lines or cancerous, we started anew with Transcripts per Million values. We selected the genes expressed in at least 50% of the samples, performed log2 and quantile normalisation and corrected for the same covariates as

before. We then performed a new principal component analysis (PCA) and used the components in a multinominal logistic regression model trained on the known sample annotations.

One major confounder with tissue type is the associated study. Typically, samples from the same study are sequenced using the same type of sequencer and read length, and most studies investigate a single tissue. But there are many differences among the different studies. We can correct for these to some extent by including technical differences as confounders, but we found that this adversely affected our prediction accuracy. We therefore devised the following strategy to create a representative training set. Ideally, we would only use a single sample per tissue from each study to train the prediction model. In practise, for some tissues, this would result in a rather limited number of usable samples. To overcome this, we increased the number of samples per tissue per study to ensure at least 50 training samples per tissue. Based on early tests, we noticed that we could not reliably discriminate between adipose and breast samples. These samples were therefore combined in a single adipose-breast network that we refer to as a 'breast' network in this manuscript for clarity.

We then used the R package *glmnet* [2] to do lasso regression with cross validation to select an optimal lambda. This model was then applied to all samples, and we assigned each sample the tissue with the highest posterior probability. Samples for which the highest posterior probability was less then 0.5 were excluded.

As a final QC, we performed a PCA per tissue and excluded outliers. This resulted in 46,410 samples. Per tissue, we eventually used VST [3] for the normalisation and corrected the data for the covariates. A SVD was used to extract the eigenvectors with gene loadings that are used by Downstreamer for the gene prioritisation.

For the recount3 multi-tissue network, we used quantile normalisation and covariate correction for the 46,410 samples for which we have a predicted tissue assignment. Here we used SVD to obtain the eigenvectors.

1. Fehrmann, R. S. N. *et al.* Gene expression analysis identifies global gene dosage sensitivity in cancer. *Nat. Genet.* **47**, 115–125 (2015).
2. Friedman, J. H., Hastie, T. & Tibshirani, R. Regularization Paths for Generalized Linear Models via Coordinate Descent. *J. Stat. Softw.* **33**, 1–22 (2010).
3. Love, M. I., Huber, W. & Anders, S. Moderated estimation of fold change and dispersion for RNA-seq data with DESeq2. *Genome Biol.* **15**, 550 (2014).

Fig. S1. Number of cancer-specific drivers and the number shared across tumour types

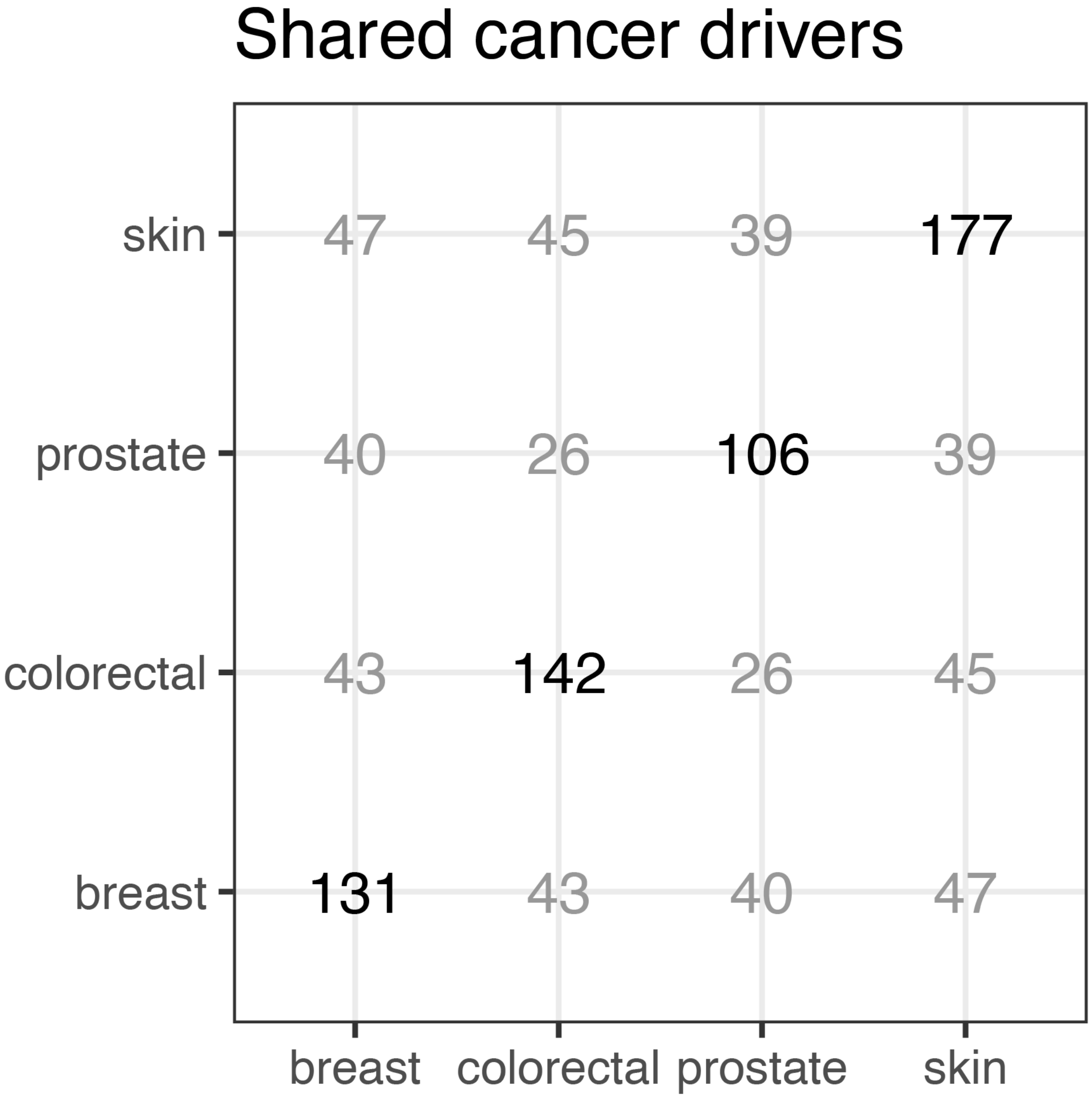

Fig. S2. Enrichment of cancer-specific driver genes for different PascalX prioritisations

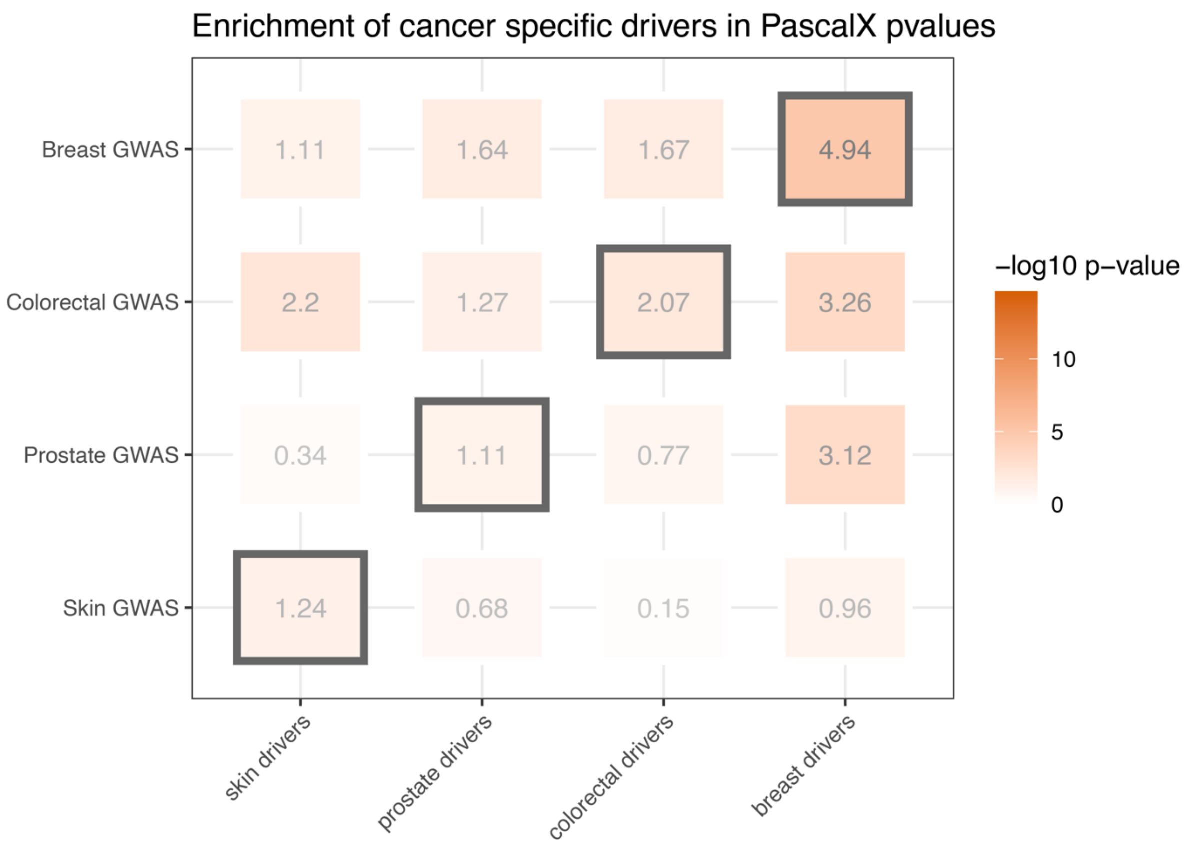

Fig. S3. Enrichment of cancer-specific somatic and germline driver genes for different multi-tissue- and tissue-specific-based gene-prioritisation methods applied to cancer GWAS summary statistics

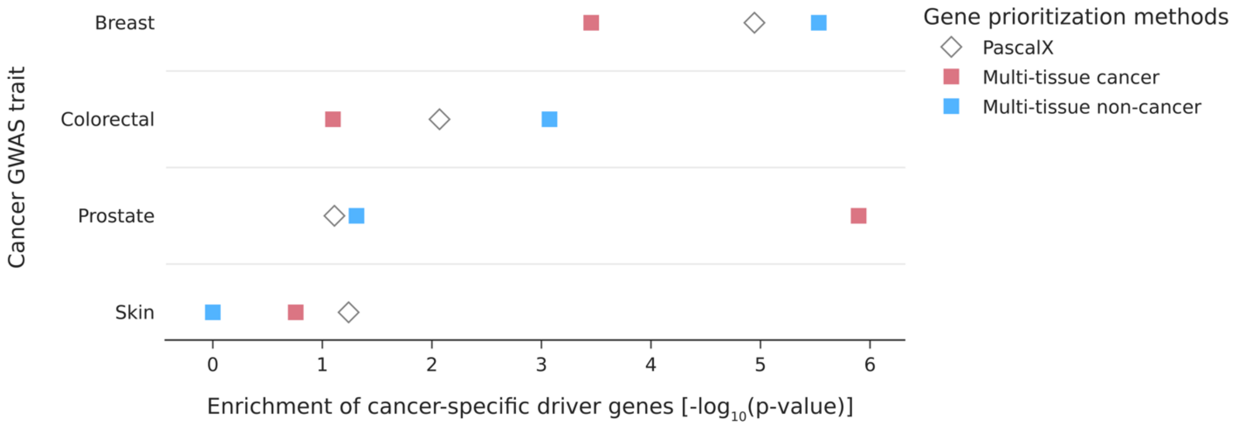

Fig. S4. Enrichment of cancer-specific driver genes for different tissue-specific matched networks

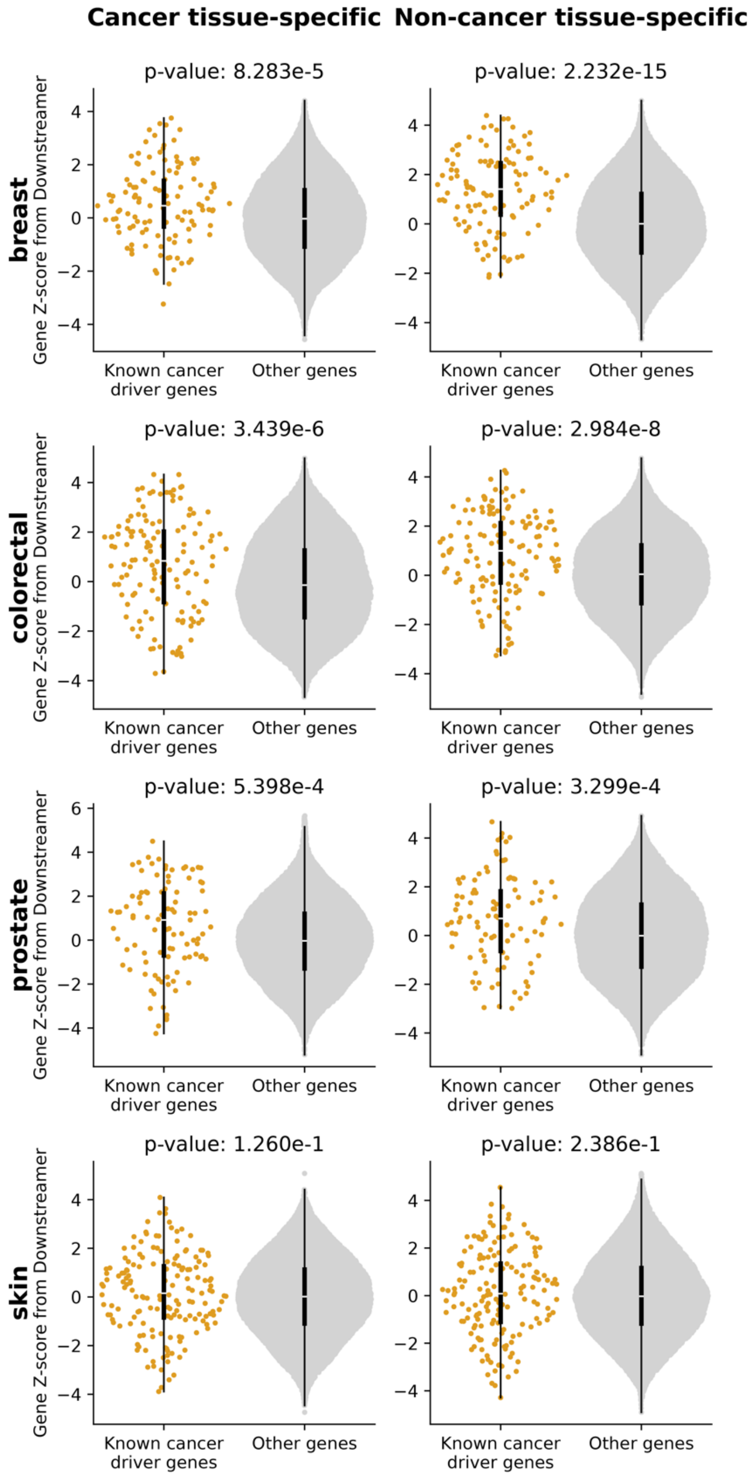

Fig. S5. Enrichment of cancer-specific somatic driver genes for all combinations of different multi-tissue and tissue-specific gene-prioritisation methods applied to cancer GWAS summary statistics using PoPS.

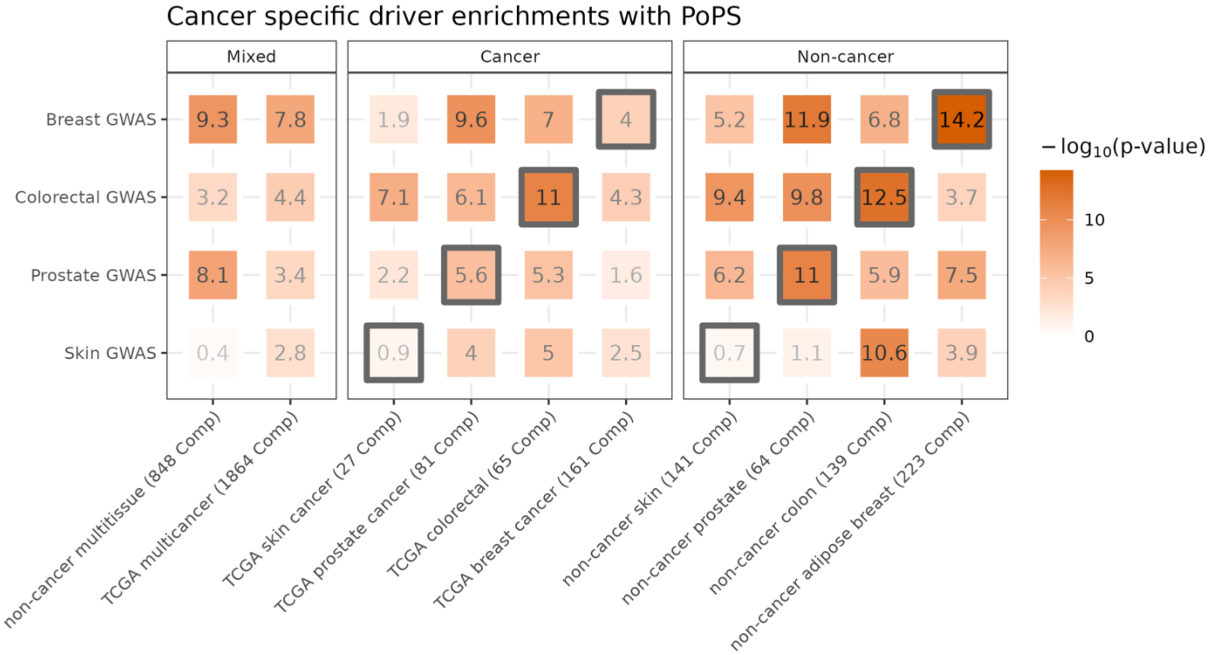

Fig. S6. Enrichment of COSMIC tier 1 cancer-specific driver genes for different tissue-specific matched networks

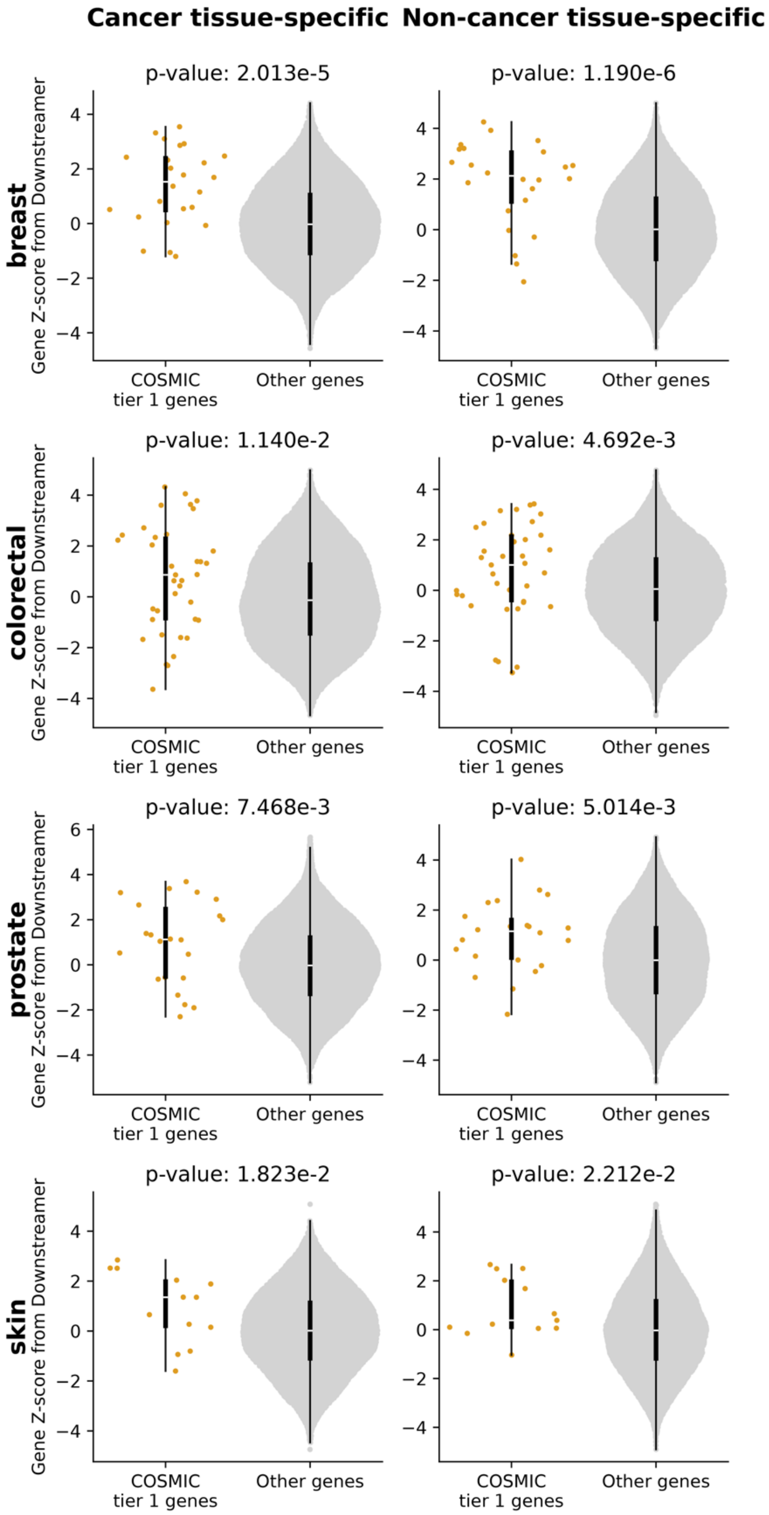

Fig. S7. Enrichment of cancer drivers suspected to be tumour suppressors

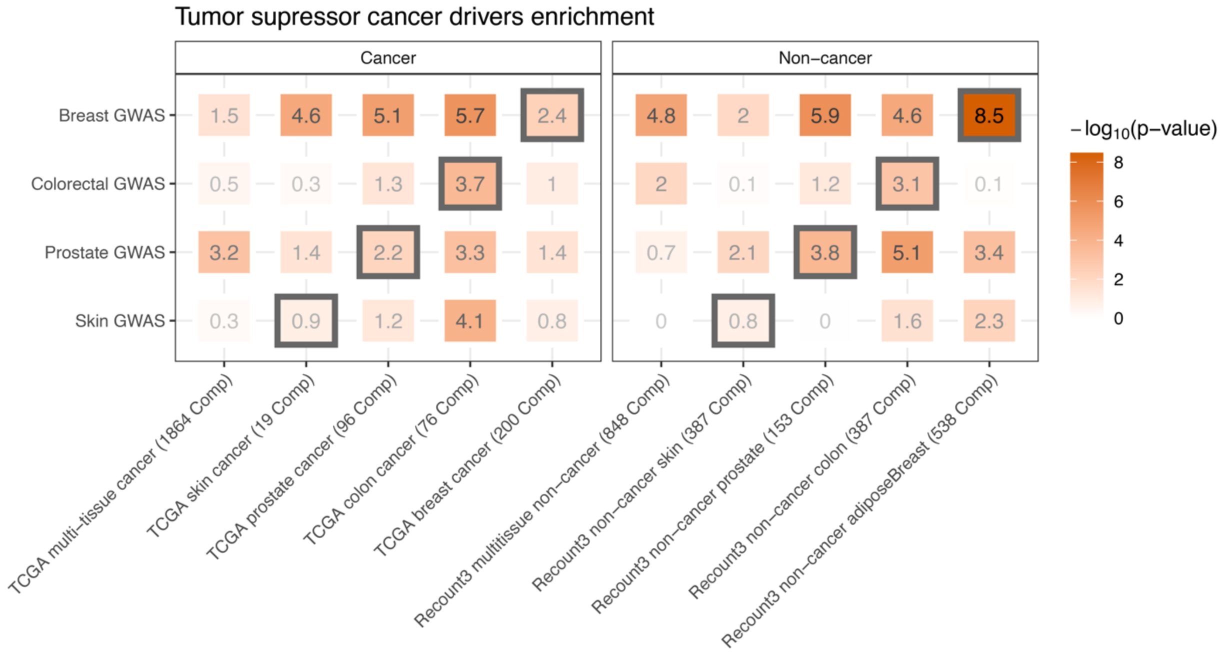

Fig. S8. Enrichment of cancer drivers suspected to be oncogenes

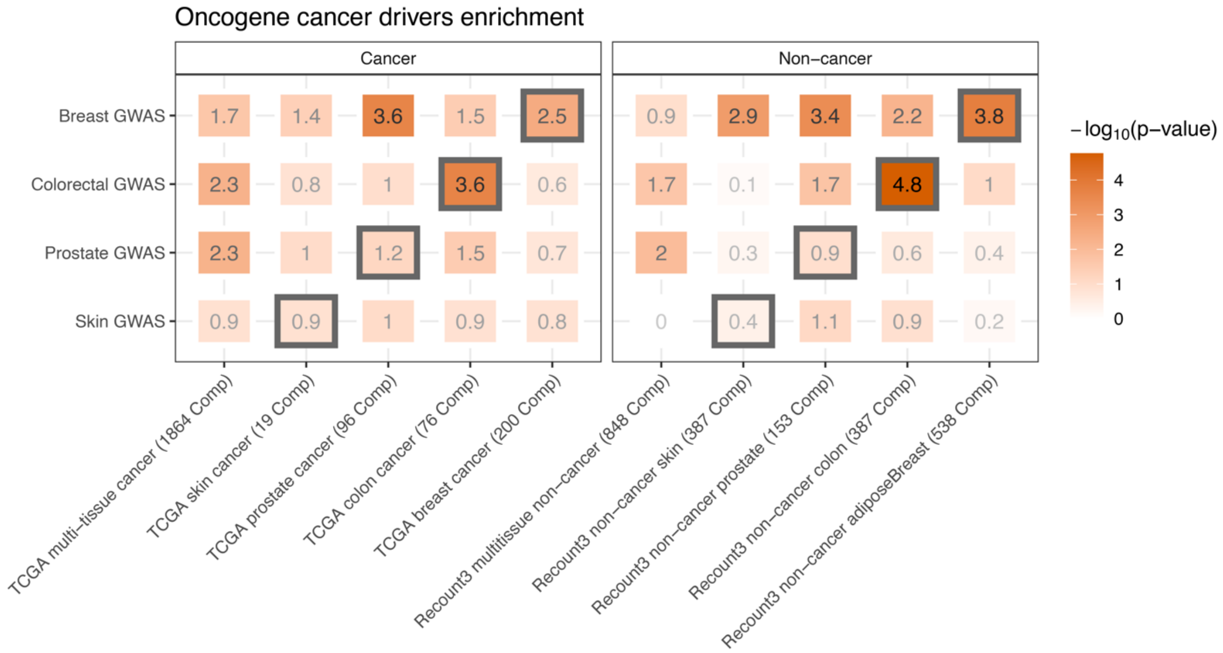

Fig. S9. Enrichment of cancer drivers exclusive to their tissue of origin for the different prioritisation scores

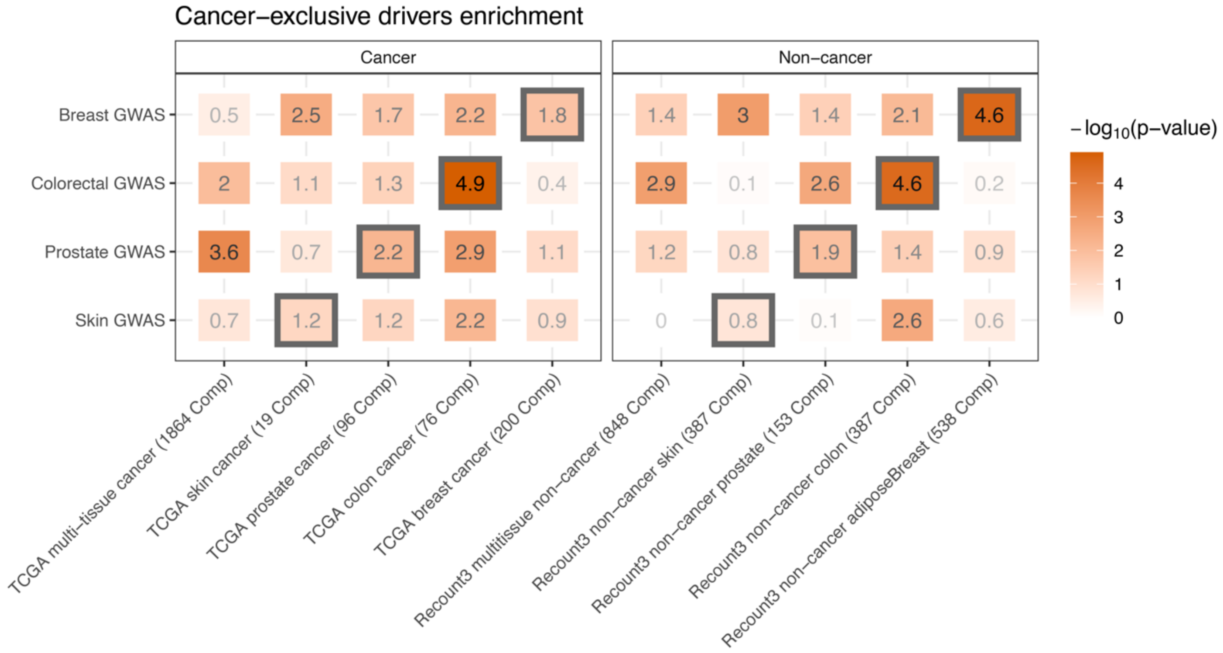

Fig. S10. Correlations of Downstreamer Z-scores of all combinations of different multi-tissue and tissue-specific networks

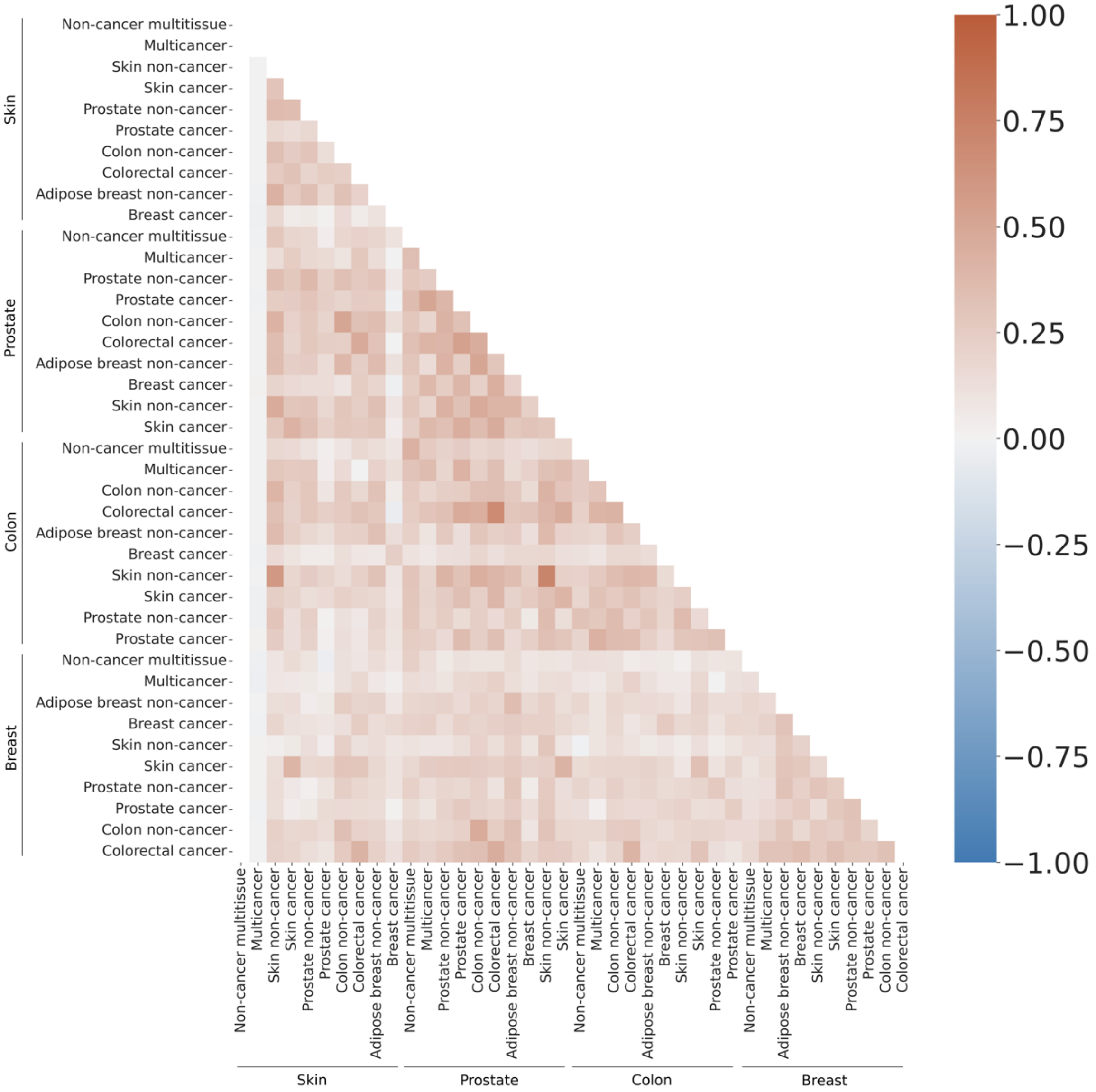

Fig. S11. Downstreamer gene-prioritisation scores from multi-tissue networks versus loss-of-function Z-scores indicating gene constraint

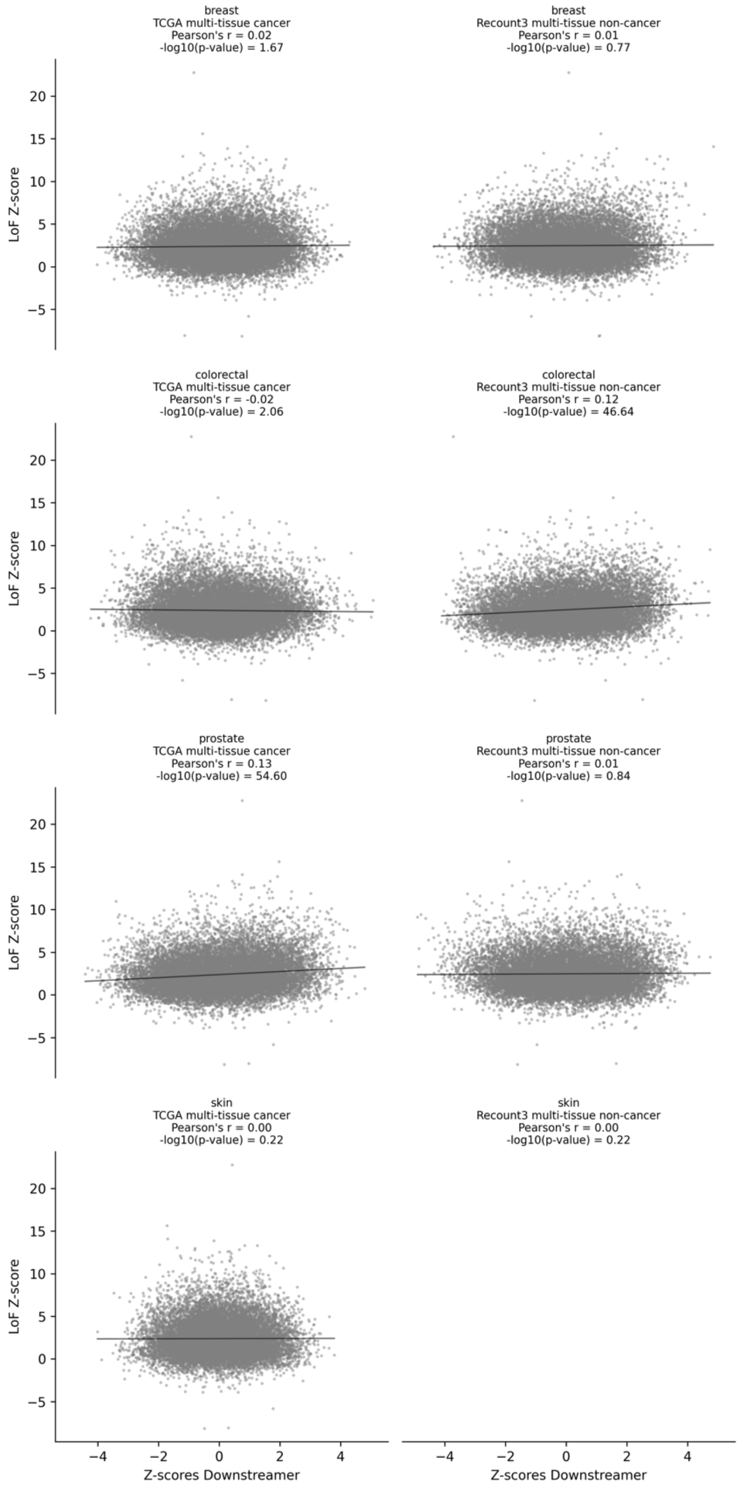

Fig. S12. ***Downstreamer gene-prioritisation scores from tissue-specific networks versus loss-of-function Z-scores indicating gene constraint***

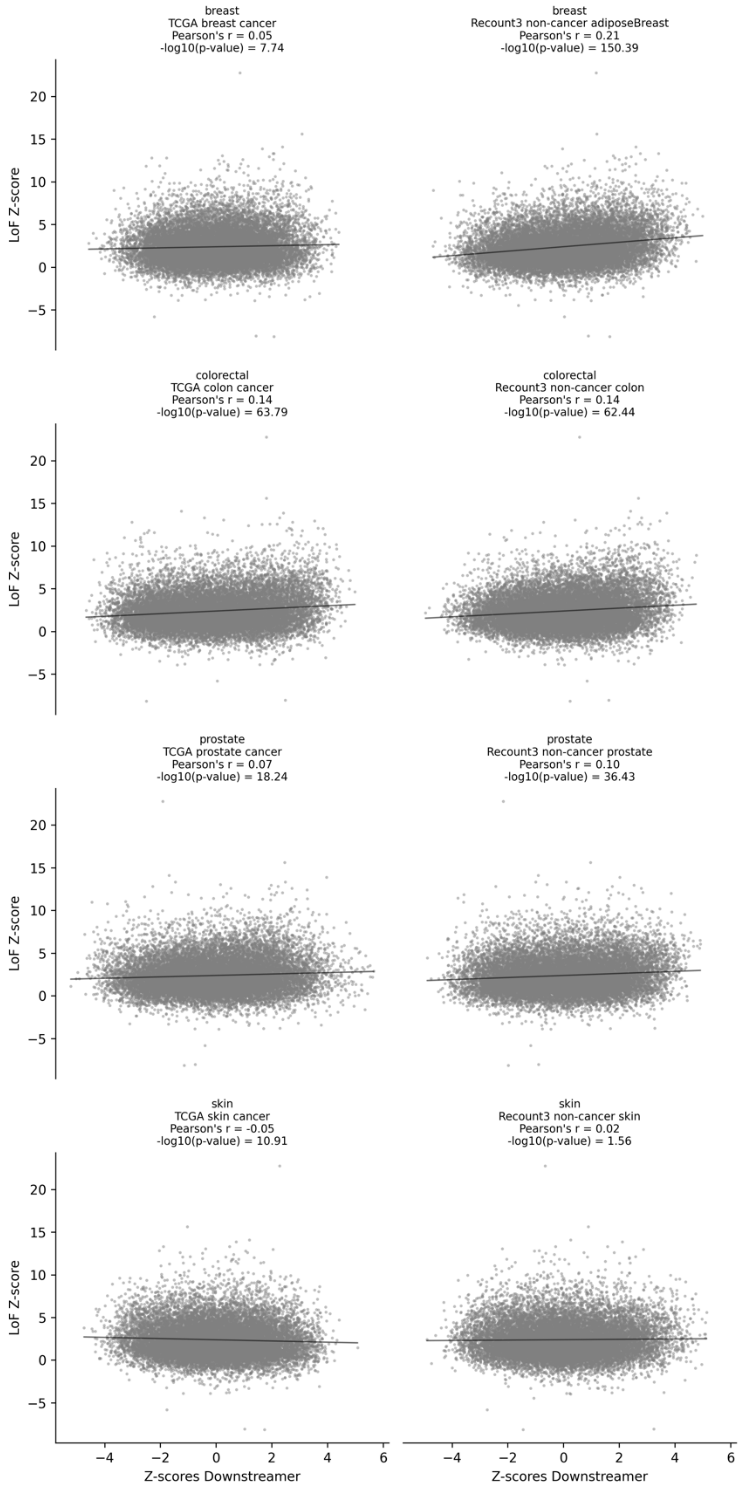

Provided separately

Table S1. All GWAS of cancer traits considered in this study

Table S2. Somatic cancer driver genes

Table S3. PascalX scores

Table S4. Downstreamer gene prioritisation

Table S5. Gene linkage disequilibrium scores

Provided separately

Data S1. Downstreamer gene prioritization (flat text)

Data S2. Pathway analysis
